# Human Lymph Node Stromal Cells Have the Machinery to Regulate Peripheral Tolerance during Health and Rheumatoid Arthritis

**DOI:** 10.3390/ijms21165713

**Published:** 2020-08-09

**Authors:** Janine S. Hähnlein, Reza Nadafi, Tineke A. de Jong, Johanna F. Semmelink, Ester B. M. Remmerswaal, Mary Safy, Krijn P. van Lienden, Mario Maas, Danielle M. Gerlag, Paul P. Tak, Reina E. Mebius, Heidi Wähämaa, Anca I. Catrina, Lisa G. M. van Baarsen

**Affiliations:** 1 Department of Rheumatology & Clinical Immunology and Department of Experimental Immunology, Amsterdam Infection & Immunity Institute, Amsterdam UMC, University of Amsterdam, Meibergdreef 9, 1105 AZ Amsterdam, The Netherlands; jhaehnlein@hotmail.com (J.S.H.); t.a.dejong@amsterdamumc.nl (T.A.d.J.); j.fsemmelink@amsterdamumc.nl (J.F.S.); m.safy@umcutrecht.nl (M.S.); dmgerlag@gmail.com (D.M.G.); tak.paulpeter@gmail.com (P.P.T.); 2 Amsterdam Rheumatology & Immunology Center (ARC), Academic Medical Center, 1105 AZ Amsterdam, The Netherlands; 3 Department of Molecular Cell Biology and Immunology, Amsterdam UMC, VU Medical Center, Vrije Universiteit Amsterdam, 1081 HZ Amsterdam, The Netherlands; r.nadafi@amsterdamumc.nl (R.N.); r.mebius@amsterdamumc.nl (R.E.M.); 4 Department of Immunology, Leiden University Medical Center, 2333 ZA Leiden, The Netherlands; 5 Renal Transplant Unit, Division of Internal Medicine and Department of Experimental Immunology, Amsterdam Infection & Immunity Institute, Amsterdam UMC, University of Amsterdam, Meibergdreef 9, 1105 AZ Amsterdam, The Netherlands; b.m.remmerswaal@amsterdamumc.nl; 6 Department of Radiology, Amsterdam UMC, University of Amsterdam, 1105 AZ Amsterdam, The Netherlands; k.p.vanlienden@amc.uva.nl (K.P.v.L.); m.maas@amc.uva.nl (M.M.); 7 Kintai Therapeutics, Cambridge, MA 02140, USA; 8 Internal Medicine, Cambridge University, Cambridge, CB2 1TN, UK; 9 Rheumatology, Ghent University, 9000 Ghent, Belgium; 10 Rheumatology Unit, Department of Medicine, Karolinska University Hospital and Karolinska Institutet, 17176 Stockholm, Sweden; heidi.wahamaa@ki.se (H.W.); anca.catrina@ki.se (A.I.C.)

**Keywords:** lymph node stromal cells, rheumatoid arthritis, tolerance, autoimmunity

## Abstract

Background: In rheumatoid arthritis (RA) the cause for loss of tolerance and anti-citrullinated protein antibody (ACPA) production remains unidentified. Mouse studies showed that lymph node stromal cells (LNSCs) maintain peripheral tolerance through presentation of peripheral tissue antigens (PTAs). We hypothesize that dysregulation of peripheral tolerance mechanisms in human LNSCs might underlie pathogenesis of RA. Method: Lymph node (LN) needle biopsies were obtained from 24 RA patients, 23 individuals positive for RA-associated autoantibodies but without clinical disease (RA-risk individuals), and 14 seronegative healthy individuals. Ex vivo human LNs from non-RA individuals were used to directly analyze stromal cells. Molecules involved in antigen presentation and immune modulation were measured in LNSCs upon interferon γ (IFNγ) stimulation (*n* = 15). Results: Citrullinated targets of ACPAs were detected in human LN tissue and in cultured LNSCs. Human LNSCs express several PTAs, transcription factors autoimmune regulator (AIRE) and deformed epidermal autoregulatory factor 1 (DEAF1), and molecules involved in citrullination, antigen presentation, and immunomodulation. Overall, no clear differences between donor groups were observed with exception of a slightly lower induction of human leukocyte antigen-DR (HLA-DR) and programmed cell death 1 ligand (PD-L1) molecules in LNSCs from RA patients. Conclusion: Human LNSCs have the machinery to regulate peripheral tolerance making them an attractive target to exploit in tolerance induction and maintenance.

## 1. Introduction

Rheumatoid arthritis (RA) is a debilitating inflammatory autoimmune disease hallmarked by disease-specific autoantibody production against citrullinated proteins, but the underlying etiopathogenesis remains largely unknown [1,2]. Citrullination is a post-translational modification changing arginine side chain residues to citrulline, thereby altering structure and charge of the protein. This process occurs regularly under homeostatic conditions like apoptosis of cells where high levels of calcium activate the peptidylarginine deiminase (PADI) enzymes catalyzing citrullination. PADI activity is also detected in a wide range of inflammatory tissues [3] including RA synovial tissue where high expression levels of PADI2 and PADI4 enzymes have been reported [4]. However, *anti-citrullinated protein antibodies* (ACPAs) can be present years before the actual onset of clinical disease [5], while synovial inflammation seems absent [6,7] during this pre-clinical RA-risk phase [8]. Therefore, breaking of tolerance against citrullinated proteins is probably generated at an extra-articular site like lymphoid organs.

Tolerance by negative selection, anergy, or by generation of regulatory T cells (T_regs_) is induced during lymphocyte maturation in thymus and maintained in the periphery. Through presentation of peripheral tissue antigens (PTAs) by medullary thymic epithelial cells (mTECs) in the thymus, self-reactive thymocytes are deleted or become unresponsive [9]. Unsurprisingly, loss of expression of these PTAs, which is driven by the transcription factors autoimmune regulator (AIRE), deformed epidermal autoregulatory factor 1 (DEAF1), and FEZ family zinc finger 2 (Fezf2) [10,11,12,13], leads to autoimmunity [10,12,14]. In humans, where AIRE expression is observed in the thymus and in dendritic cells (DCs) [15,16], AIRE mutations cause a multi-systemic autoimmune syndrome, known as autoimmune polyendocrinopathy-candidiasis-ectodermal dystrophy (APECED) [17].

Some self-reactive lymphocytes escape the thymic negative selection and are present in healthy individuals [18]. Safeguarding tolerance in the periphery is therefore crucial and studies in mice show that lymph node (LN) stromal cells (LNSCs) have therein a dominant role. LNSCs possess an impressive arsenal to shape T and B cell responses for maintenance of the delicate balance between tolerance and appropriate immune response [19,20]. Several subsets of LNSCs have been described, and although the number of subsets is expanding, six subsets are well defined according to their function, location within the LN, and the expression of surface markers podoplanin (PDPN, gp38) and CD31 (PECAM-1): fibroblastic reticular cells (FRCs: CD31− gp38+), follicular dendritic cells (FDCs: CD31− gp38+/−), marginal reticular cells (MRCs: CD31− gp38+/−), the rather poorly studied double negative cells (DNs: CD31− gp38−), lymphatic endothelial cells (LECs: CD31+ gp38+), and blood endothelial cells (BECs: CD31+ gp38−) [21,22]. Among others, FDCs and LECs serve as antigen libraries since they catch, preserve, and present antigens over longer periods, thereby enhancing T cell memory [23,24]. FRCs and LECs have the ability to limit T cell proliferation during ongoing inflammation by secretion of nitric oxide (NO) and expression of other negative regulators such as indoleamine 2,3-dioxygenase (IDO) to protect LN integrity and to contract immune responses for return to steady state [25,26]. Furthermore, studies have convincingly demonstrated that several LNSC subsets present PTAs on major histocompatibility complex (MHC) class I and induce clonal deletion [10,11,27,28]. Additionally, CD4+ T cells can be tolerized via PTA presentation on MHC class II or by presentation of MHC-II-peptide complexes acquired from DCs [29,30]. Moreover, expression and subsequent presentation of PTAs by LNSC in the context of MHC class II to CD4+ T cells can also lead to maintenance of T_regs_ [31]. Moreover, recently we demonstrated that LNSCs convert naïve autoreactive CD4+ T cells into antigen-specific T_regs_ cells and suppress autoreactive T follicular helper (T_fh_) and B cells responses [32].

Taking into account the tremendous influence of LNSCs on peripheral tolerance and lymphocyte regulation we hypothesize that malfunctioning of LNSCs might lead to a microenvironment causing loss of tolerance and autoantibody production. In this study we investigated for the first time in humans whether the LN is a potential place where citrullination of RA-related PTAs occurs and whether human LNSCs, like murine LNSCs, exhibit the tools for tolerance induction. Finally, we compared the expression of citrullinated proteins, PTAs, and immunomodulatory molecules on human LNSCs of healthy individuals to LNSCs from RA patients and autoantibody positive individuals at risk of developing RA (RA-risk individuals). Our data reveal that human LNSCs express citrullinated proteins targeted by ACPAs and are well equipped to regulate (RA-related) tolerance.

## 2. Results

### 2.1. Citrullinated Antigens Targeted by ACPAs Are Present in Human LN Tissue and in Cultured LNSCs

**Figure 1:**
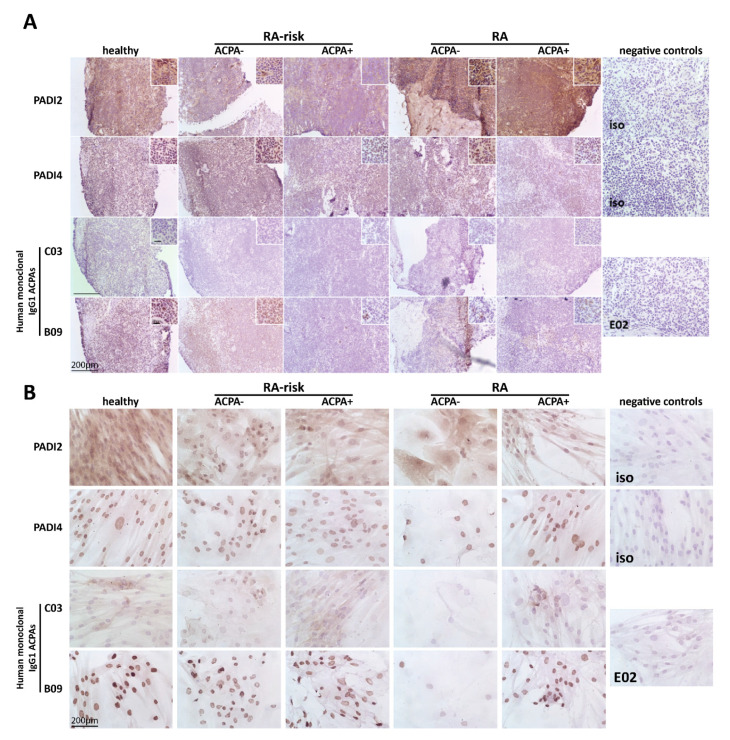
Expression of peptidylarginine deiminases (PADIs) and citrullinated proteins targeted by anti-citrullinated protein antibodies (ACPAs) in lymph node (LN) tissue and cultured lymph node stromal cells (LNSCs). (**A**) LN tissue was stained for PADI2 and PADI4 proteins and citrullinated proteins targeted by ACPAs using human monoclonal antibodies C03 and B09. Staining with isotype control (iso) (healthy LN tissues for PADI2 and PADI4 are represented) or control antibody E02 (rheumatoid arthritis (RA) ACPA+ patients LN tissue is represented) was negative. A representative picture of each donor group of the immunohistochemical analysis in LN tissue is displayed (*n* = 3 per donor group; healthy individuals, RA-risk ACPA− individuals, RA-risk APCA+ individuals, RA ACPA− patients, and RA ACPA+ patients). (**B**) Cultured LNSCs were stained against PADI2 and PADI4 protein and citrullinated proteins targeted by ACPAs using human monoclonal antibodies C03 and B09. Staining with isotype controls (iso; RA-risk ACPA+ individuals LNSCs for PADI2 and healthy individuals LNSCs for PADI4 are represented) and control antibody E02 (RA ACPA+ patients LNSCs is represented) was negative. A representative picture of each donor per group of the immunohistochemical analysis in cultured LNSCs is displayed (*n* = 3 per donor group; healthy individuals, RA-risk ACPA− individuals, RA-risk APCA+ individuals, RA ACPA− patients and RA ACPA+ patients, passages 3–11). The larger bar in the left corner of images represents 200 µm and the smaller bar inside the magnified images in (**A**) represents 20 µm.

First we investigated by immunohistochemistry the presence of PADI enzymes required for citrullination in LN tissue and cultured LNSCs of a small cohort of individuals (healthy individuals, RA-risk ACPA− individuals, RA-risk ACPA+ individuals, RA ACPA− patients, and RA ACPA+ patients; for each subgroup *n* = 3, total *n* = 15). Both PADI2 and PADI4 enzymes were abundantly present in LN tissue. In cultured LNSCs, PADI2 and PADI4 enzymes were detected intracellularly with PADI4 showing a nuclear expression pattern as reported before [33] (Figure 1A,B, upper panels). Using unique monoclonal ACPAs isolated from RA patients’ synovial fluid, which are cross-reactive to one or more citrullinated antigens [34,35], we next analyzed the presence of RA-associated citrullinated proteins in LN tissues and cultured LNSCs. Staining with monoclonal ACPAs C03 and B09 revealed the presence of ACPA-targets in LN tissue as well as in cultured LNSCs derived from all study groups (Figure 1A,B, lower panels). Overall, each antibody showed a distinct staining pattern with variable intensity between donors tested. C03-binding was only detected in a few donors, while B09 was highly abundant in most donors tested, with expression mainly restricted to nuclei. These data show that citrullinated proteins, targeted by ACPAs, are present in LN tissue and cultured LNSCs, and at similar levels in healthy individuals, RA-risk individuals, and RA patients.

### 2.2. Cultured Human LNSCs Express the Transcription Factors AIRE and DEAF1

**Figure 2:**
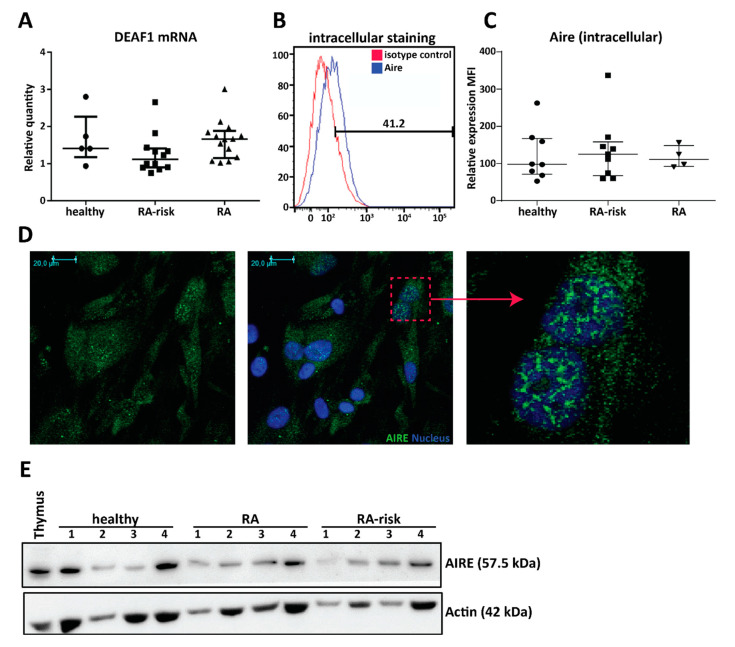
Expression of the peripheral tissue antigen (PTA) driving transcription factors autoimmune regulator (AIRE) and deformed epidermal autoregulatory factor 1 (DEAF1) in human LNSCs. (**A**) Expression of DEAF1 in cultured LNSCs of passage 2 was assessed by qPCR and compared between different donor groups (healthy individuals *n* = 5, RA-risk individuals *n* = 12 and RA patients *n* = 14). Relative quantity is displayed as median and interquartile range. (**B**) Intracellular expression of AIRE protein in cultured LNSCs was measured by flow cytometry. Histograms presenting % of positive cells in comparison to isotype staining. (**C**) The scatter plot represents the mean fluorescence intensity (MFI) of intracellular AIRE expression in cultured human LNSCs (passages 3–5) from individuals in different donor groups (healthy individuals *n* = 8, RA-risk individuals *n* = 9 and RA patients *n* = 4). Relative quantity is presented as median and interquartile range. (**D**) Representative pictures of immunofluorescence staining combined with confocal microscopy displaying AIRE (green) and nucleus (blue) in LNSCs (RA patient; passage 3) cultured on chambers slides. Isotype controls were negative. (**E**) Western blot analysis of AIRE protein expression in cultured LNSCs of 12 donors (passages 4–8) is shown. Actin was used as loading control and thymic tissue was used as positive control for AIRE expression.

The expression of an abundant number of PTAs is regulated by transcription factor AIRE [17] and DEAF1 [10,11]. In culture expanded LNSCs (all passage 2) DEAF1 mRNA was strongly expressed at similar levels in all donor groups (Figure 2A, healthy individuals *n* = 5, RA-risk individuals *n* = 12, and RA patients *n* = 14). In contrast, AIRE mRNA was very low but detected by qPCR while AIRE protein expression was easily measured by flow cytometry (Figure 2B,C), immunohistochemistry (Figure 2D), and Western blot (Figure 2E). Mean fluorescence intensity (MFI) of AIRE expression (intracellular) in cultured LNSCs (passages 3–5) was comparable between healthy individuals, RA-risk individuals, and RA patients (Figure 2C, healthy individuals *n* = 5, RA-risk individuals *n* = 9 and RA patients *n* = 4). Moreover, comparing intracellular versus intranuclear staining revealed most AIRE protein within the nucleus, which was confirmed by immunohistochemistry (Figure 2D and Figure S1). Additionally, also by Western blot, we observed that AIRE protein was ubiquitously expressed in cultured LNSCs from healthy individuals, RA-risk individuals, and RA patients (Figure 2E) and that the protein band in LNSCs corresponded to the AIRE expression observed in thymus tissue. Overall, these data show that both AIRE and DEAF1 are present in cultured human LNSCs.

### 2.3. Variable Expression of Disease-Related PTAs in Human LNSCs

**Figure 3:**
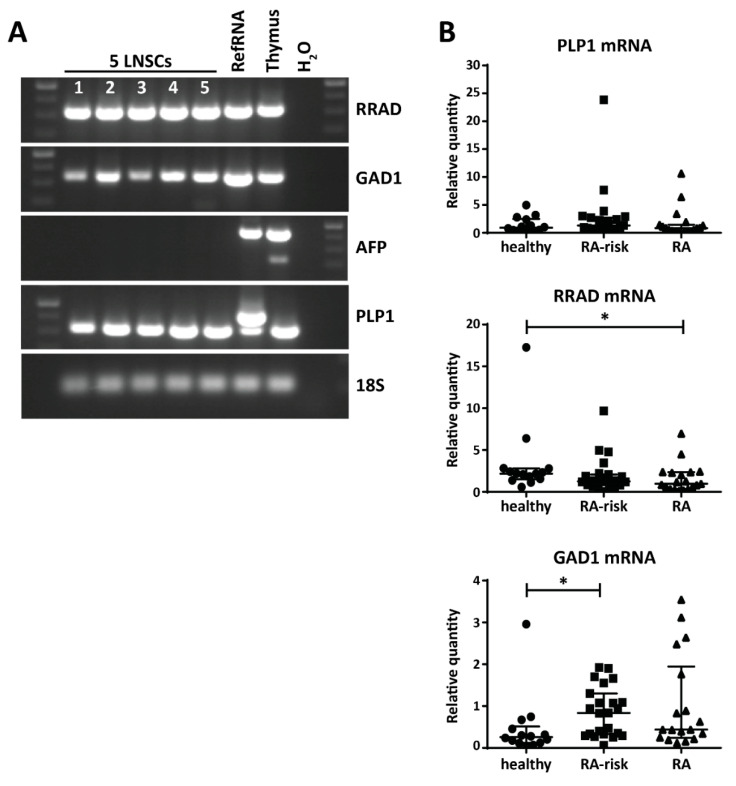
Expression of disease-related PTAs in human LNSCs. (**A**) mRNA expression of Ras Related Glycolysis Inhibitor and Calcium Channel Regulator (RRAD), Glutamate Decarboxylase 1 (GAD1), Proteolipid Protein 1 (PLP1), and Alpha Fetoprotein (AFP) in five LNSC individuals (1: healthy individuals passage 4; 2: RA-risk individuals passage 4; 3: RA patients passage 4; 4: RA patients passage 4; 5: RA patients passage 3) analyzed by PCR and visualized on agarose gel. cDNA prepared from an arbitrary in house-made RNA sample pool containing all human tissues (RefRNA) as well as a human thymus sample served as positive control and H_2_O in exchange for cDNA as negative control. (**B**) Expression of PLP1, RRAD, and GAD1 and was assessed by qPCR in different donor groups (healthy individuals *n* = 14, RA-risk individuals *n* = 23, and RA patients *n* = 24, all passage 2). Relative quantity is displayed as median and interquartile range. Differences between donor groups were assessed by Kruskal–Wallis followed by a post Dunn’s test. * *p* < 0.050.

Given that we found the presence of the transcription factors potentially driving PTA expression in LNSCs, we next investigated the expression levels of several disease-related PTAs. We selected PTAs previously reported to be expressed by mouse LNSCs [27,28] and involved in human diseases: Ras Related Glycolysis Inhibitor and Calcium Channel Regulator (RRAD), Glutamate Decarboxylase 1 (GAD1), Proteolipid Protein 1 (PLP1), and Alpha Fetoprotein (AFP). RRAD is implicated in several cancers [36] and plays an important role in type II diabetes [37]. GAD1 is a major autoantigen in type I diabetes and highly expressed in brain and pancreas [38]. PLP1 encodes the most abundant myelin protein which is a main target of autoreactive T cells in multiple sclerosis (MS) and is detected in cervical LNs of MS patients [39]. AFP levels serve as diagnostic marker of liver injury such as hepatocellular carcinoma or Hepatitis C infection [40]. With the exception of AFP, all these disease-related PTAs were expressed by human LNSCs (passages 3 and 4) (Figure 3A). While we did not observe a significant difference in PLP1 expression by LNSCs from different donor groups, we interestingly found that GAD1 and RRAD has a variable expression pattern in LNSCs (all passage 2) derived from healthy individuals, RA-risk individuals and RA patients (healthy individuals *n* = 14, RA-risk individuals *n* = 23, and RA patients *n* = 24). RRAD was significantly expressed at a lower level in LNSCs of RA patients (*p* = 0.037), whereas GAD1 was highly expressed in LNSCs of RA-risk individuals (*p* = 0.0129) when compared to healthy individuals (Figure 3B). Furthermore, after stratification according to ACPA status, the difference in PTA expression was especially clear in ACPA negative RA patients with almost no RRAD expression in LNSCs from ACPA negative RA patients (Figure S2). The expression of these PTAs did not correlate with any other clinical parameters such as age, gender, or antibody titers.

### 2.4. MHC Class II Expression by Human LNSC In Vitro

**Figure 4:**
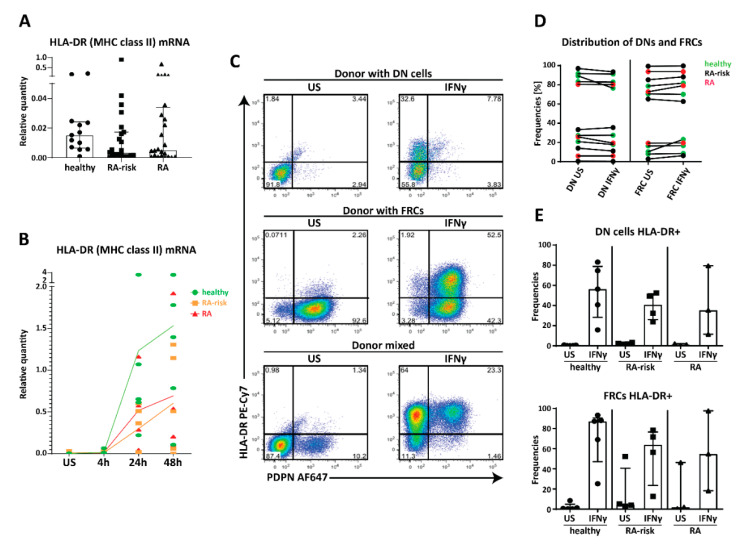
Expression of human leukocyte antigen-DR (HLA-DR) (major histocompatibility complex (MHC) class II) in cultured human LNSCs. (**A**) Expression of HLA-DR mRNA in cultured human LNSCs (all passage 2) from healthy individuals, RA-risk individuals, and RA patients. (**B**) Induction of HLA-DR was assessed by qPCR after stimulation with interferon γ (IFNγ) at different time points (unstimulated (US), 4, 24, and 48 h, all passage 2). Data are represented as relative quantity in each donor (dot) measured over time, where the line indicates the median expression level within one study group over time. (**C**) Flow cytometry gating strategy used to identify CD45− stromal cells according to their Podoplanin (PDPN) and HLA-DR (MHC class II) expression. Gating was based on negative isotype staining. FACS plots display cultured LNSCs from three representative donors (double negative (DN) cells derived from RA-risk individuals passage 5, fibroblastic reticular cells (FRCs) from RA patients passage 7, and LNSCs from healthy individuals passage 5 containing DN and FRCs) out of 13 individuals tested (healthy individuals *n* = 5, RA-risk individuals *n* = 5, and RA patients *n* = 3). Dot blot shows their expression profile under unstimulated condition versus stimulation for 72 h with IFNγ. Numbers adjacent to the outlined areas indicate percentage of cells in the gated population. (**D**) Frequencies of DNs and FRCs per donor under unstimulated conditions and stimulated for 72 h with IFNγ is shown. (**E**) The frequencies of HLA-DR positive cells after stimulation for 72 h with IFNγ in FRCs (PDPN+) and DN cells (PDPN−) are depicted in two separate graphs. Data are represented as median with interquartile range.

Since direct antigen presentation is very difficult to study in human LNSCs due to MHC restriction and lack of high number of antigen-specific T cells recognizing PTAs, we aimed to study the machinery needed for antigen presentation and lymphocyte modulation in cultured LNSCs. Under homeostatic conditions the human leukocyte antigen-DR (HLA-DR) gene participating in the MHC class II complex, is expressed by cultured human LNSCs (all passage 2) at very low levels in all donor groups tested (Figure 4A). Of interest, a non-significant lower expression of HLA-DR was observed in cultured LNSCs obtained from RA-risk individuals and RA patients. We next used interferon γ (IFNγ) to study regulation of MHC class II as previously reported in mice and humans [26,29]. HLA-DR mRNA was strongly increased after 24 and 48 h of stimulation, when compared to unstimulated (US), with no significant differences between donor groups, though induction was highly variable between donors (Figure 4B, *n* = 5 per donor group). On protein level, HLA-DR was strongly increased after 72 h of IFNγ stimulation (Figure 4C,D). Interestingly, next to FRCs (Podoplanin+, CD31−) also DNs (Podoplanin−, CD31−) clearly upregulated MHC class II. Podoplanin expression was not influenced by IFNγ stimulation (Figure 4C). Furthermore, the number of FRCs and DNs was similar between donor groups (Figure 4D) and within these two subsets HLA-DR expression was similarly increased in all donor groups although a large inter-donor variation in induction was observed (Figure 4E, healthy individuals *n* = 5, RA-risk individuals *n* = 4, and RA patients *n* = 3). These findings did not correlate with clinical parameters such as age, gender, or antibody titers. Overall, these data suggest that human FRCs as well as DNs are equipped to potentially present antigens to CD4+ T cells.

### 2.5. Potential Immunomodulation by Cultured Human LNSC

**Figure 5:**
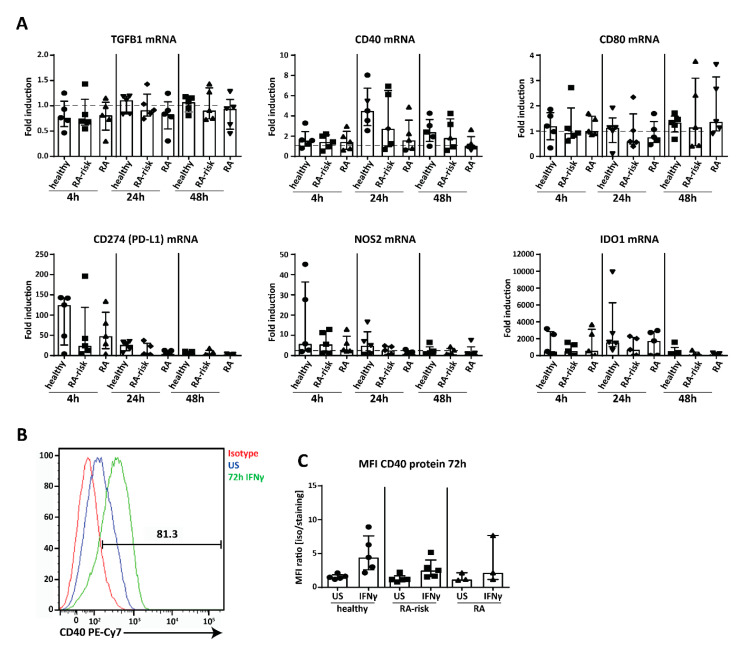
Expression of immunomodulatory molecules in cultured human LNSCs. (**A**) Induction of transforming growth factor beta 1 (TGFB1) CD40, CD80, CD274 (PD-L1; programmed cell death 1 ligand), nitric oxide synthase 2 (NOS2), and indoleamine 2,3-dioxygenase 1 (IDO1) was assessed by qPCR in cultured LNSCs (passages 4–9) after stimulation with IFNγ at different time points (4, 24, and 48 h). Data are represented as fold induction (median with interquartile range) by comparing the mRNA levels in stimulated cells to corresponding unstimulated cells in 15 donors (*n* = 5 per donor group). The dotted line represents a fold induction of 1. (**B**) CD40 protein expression was measured by flow cytometry in CD45− stromal cells. Histogram depicts the increase in staining between isotype, unstimulated and stimulated for 72 h with IFNγ and is displayed for one representative donor (RA-risk individuals passage 8) out of 13 donors tested. (**C**) Induction of CD40 protein upon stimulation with IFNγ for 72 h is further presented as Mean Fluorescent Intensity (MFI) ratio (isotype/staining) in all donors measured (healthy individuals *n* = 5, RA-risk individuals *n* = 5, and RA patients *n* = 3, passages 4–9).

Next we analyzed the expression of genes potentially involved in LNSC-mediated T cell modulation, thereby we used IFNγ, which is produced by T cells upon their activation and differentiation, as a stimulus [41]. Genes analyzed in cultured LNSCs of passages 4–9 after 4, 24, and 48 h stimulation included interferon gamma receptor 1 (IFNGR1), co-stimulatory molecules cluster of differentiation 40 (CD40), CD80 and CD86 [27,42], immunosuppressive cytokines interleukin 10 (IL-10) and transforming growth factor beta 1 (TGFB1), which are both involved in T_reg_ cell induction [43,44], and the negative T cell regulators CD274 (programmed cell death 1 ligand (PD-L1)), nitric oxide synthase 2 (NOS2) and indoleamine 2,3-dioxygenase 1 (IDO1) [25,26] (Figure 5A, healthy individuals *n* = 5, RA-risk individuals *n* = 5, and RA patients *n* = 5, passages 4–9). Under homeostatic conditions no differences between donor groups were observed. IFNGR1 was stably expressed, at similar levels in all donor groups and unaffected by stimulation (Figure S3), showing that all donor groups are equally equipped to respond to IFNγ. TGFB1 was strongly expressed under homeostatic conditions and not strongly affected by IFNγ (Figure 5A). In contrast, IL-10 was not detected by qPCR. For the co-stimulatory molecules, we found that CD40 was strongly expressed and slightly induced at 24 h, while expression of CD80 was very low and CD86 was undetectable (Figure 5A). On the other hand, the negative regulators CD274 (PD-L1), NOS2, and IDO1 strongly responded to IFNγ, with CD274 (PD-L1) and NOS2 expression peaking at 4 h and IDO1 at 24 h (Figure 5A). Furthermore, at protein level we could detect CD40 by flow cytometry on cultured human LNSCs and expression was upregulated after 72 h stimulation with IFNγ (Figure 5B) though no significant differences between donor groups were observed (passages 4–9) (Figure 5C). Finally, none of these findings reported here on mRNA or protein level correlated with any clinical parameter such as age, gender, or autoantibody titers. Overall, these data show that human LNSCs have the capacity to modulate the adaptive immunity.

### 2.6. Ex Vivo Human LNSCs Express HLA-DR and Low Level of Co-Stimulatory and Co-Inhibitory Molecules

**Figure 6:**
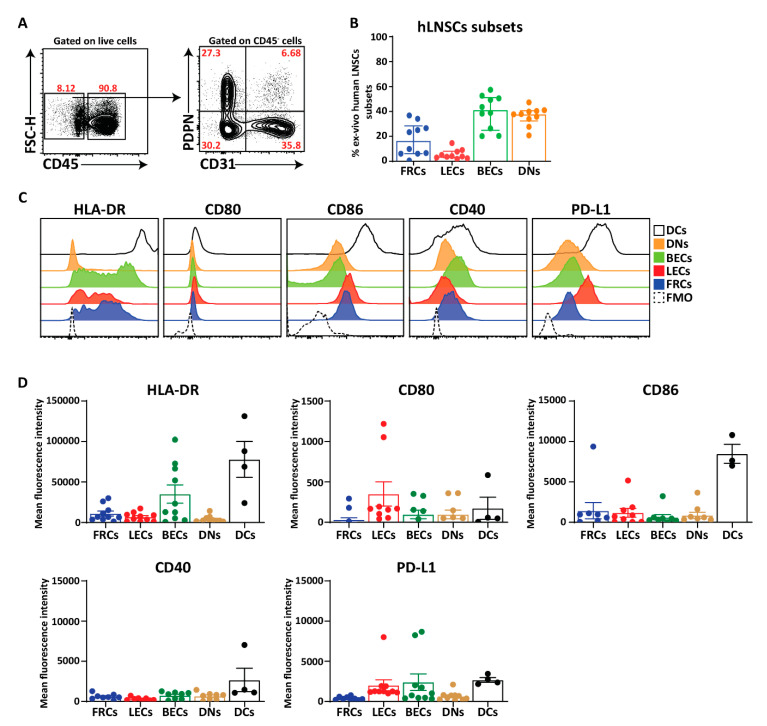
Expression of HLA-DR and co-stimulatory molecules on freshly isolated human LNSCs. (**A**) Human LNs obtained from kidney transplantation recipients were enzymatically digested, and stained for LNSC subsets based on the expression of CD31 and Podoplanin (PDPN) when gated on live CD45− cells. (**B**) Scatter plot represents the frequencies of different subsets of human LNSCs (hLNSCs) directly after digestion. (**C**) Stromal cells were gated as live CD45− cells and expression of co-stimulatory and inhibitory molecules on different subclasses of LNSCs and CD45+CD11c+ dendritic cells (DCs) were assessed using flow cytometry. Histograms show the expression of indicated molecules on different stromal cell subsets and DCs. The fluorescence minus one (FMO) was used as negative control. (**D**) Scatter plots represent mean fluorescence intensity of HLA-DR, CD80, CD86, CD40, and PD-L1 of each subset of LNSCs when gated on live CD45− cells in comparison to DCs. Data are represented as mean with SEM (*n* = 3–10 kidney recipients).

Finally, we acquired larger pieces of human LNs from kidney transplant recipients which enabled the directly ex vivo analysis of HLA-DR, co-stimulatory, and co-inhibitory molecules on human LNSCs in comparison to DCs (live CD45+CD11c+) as classical antigen presenting cells. In line with murine LNs [31,45], the human LN stromal compartment consists of four distinct subsets when gated on live CD45− cells and additional staining for CD31 and PDPN, thereby considering the PDPN+ population as one population containing FRCs, MRCs, as well as FDCs (Figure 6A,B). However, we observed more BECs and DNs in human LNs compared to mice [31,45]. HLA-DR was expressed on all subsets of human LNSCs but at different levels and considerably lower than DCs (Figure 6C,D). The MFI of HLA-DR in FRCs, LECs, and BECs was higher than DNs which is comparable to MHC-II expression described for murine LNSCs (Figure 6C,D) [31]. Similar to cultured human LNSCs (Figure 5A), co-stimulatory ligand CD80 was detected on freshly digested LNs at protein level in which the expression was relatively higher on LECs compared to other subsets. Furthermore, we could detect low levels of CD86 surface expression on different subsets of LNSCs while we were unable to detect the CD86 at mRNA level in cultured stromal cells (Figure 6C,D). CD40 expression was present on all the subsets and at lower levels than DCs (Figure 6C,D). The expression level of PD-L1+ on endothelial cells (LECs and BECs) was equivalent to DCs but lower on FRCs and DNs. These data noticeably show that ex vivo LNSCs possess HLA-DR, co-stimulatory and co-inhibitory molecules.

## 3. Discussion

Herein we demonstrate for the first time that PADI2 and PADI4 enzymes as well as citrullinated proteins targeted by ACPAs are present in human LN tissue as well as in cultured human LNSCs during health and different phases of RA. Furthermore, we show that human LNSCs express certain PTAs as well as transcription factors AIRE and DEAF1 together with molecules involved in antigen presentation and immunomodulation. Of interest, our data points towards an altered LN microenvironment in RA patients compared to healthy individuals as seen by variable expression levels of some PTAs.

Citrullination occurs during life under homeostatic conditions but increases in many tissues during inflammation [46]. With PADIs and citrullinated proteins observed in healthy individuals, ACPA-negative and ACPA-positive LN tissue, and cultured LNSCs, our data clearly reveals that citrullination is on-going in human LNs and that this process occurs in both healthy individuals and RA patients’ LNSCs. However, a deeper conclusion cannot be drawn since the antibodies used are reactive to several citrullinated targets [47] and we only investigated a small number of donors in this explorative study.

In the context of peripheral tolerance by LNSCs we confirm expression of DEAF1 and AIRE, with AIRE protein being localized primarily nuclear [11] with some cytosolic AIRE as reported in other mammalian cells [48]. Furthermore, we observed a differential expression pattern of disease-related PTAs in LNSCs of RA-risk individuals and RA patients. Of interest, these differences were especially pronounced in LNSCs derived from ACPA negative individuals, which form potentially a genetically distinct patient group [49]. Expression of RRAD was found to be significantly lower in LNSCs of RA patients compared to healthy individuals. At first glance there is no link to autoimmunity since RRAD overexpression is associated with type II diabetes [37,50], which is caused by acquired insulin resistance. However, RA is an important risk factor [51] for this type of diabetes, possibly due to RA-driven inflammation [52]. Since it has been shown that murine LNSCs can control the formation of autoreactive T cells by presenting specific PTA in the context of MHC molecules [27,32,53], it is possible to speculate that LNSCs obtained from RA patients are not capable of suppressing RRAD-specific T cells due to lower expression of this PTA. Although the limitation of our study is the low number of PTAs we analyzed. Investigating the expression of various PTAs by LNSCs from RA patients will be of interest in future studies to confirm this observation. Moreover, further experiments showing antigen presentation and tolerance induction by human LNSCs are necessary, though highly challenging to conduct. So far, we lack the knowledge on well-defined PTA presented by human LNSCs and the availability of corresponding autoreactive human T cells. However, recently a multi-tetramer assay has been developed to detect citrulline-specific T cells in both healthy individuals and RA patients [54]. These tetramers might allow us to study tolerance capacity of LNSCs in controlling citrulline-specific T cells using our in vitro model containing expanded human LNSCs.

Our data reveal that both ex vivo and in vitro, human LNSCs might have the potential to present PTAs directly as they strongly express HLA-DR especially after stimulation with IFNγ. Of interest, our study shows that human FRCs, and also DNs have equal capacity to induce HLA-DR, pointing towards potentially similar roles of these two subsets in humans [21]. However, studies in mice diverge on whether LNSCs present directly to CD4+ T cells. Two studies promoted direct presentation [31,55] and shuttling MHC class II-peptide complexes from DCs [29], while another group showed that LNSCs shuttle antigens to DCs and do not induce tolerance themselves [30]. Similarly, in humans, LECs were able to process antigens, but failed to induce allogeneic CD4+ T cell proliferation [26]. Since LNSCs needed several weeks to grow to confluence and did not contain CD45 positive hematopoietic cells such as DCs, we can conclude that human LNSCs express MHC class II themselves. Accordingly, our ex vivo analyses reveal that human LNSCs express low level HLA-DR in comparison to DCs. Furthermore, the antibody L243 used to stain MHC class II molecules detects a conformational epitope on HLA-DRαβ which depends on the peptide-loading and consequent correct folding of the αβ heterodimer [56], indicating the presence of functional MHC class II molecules containing peptides on the LNSC cell membrane. Nonetheless, this culture technique for human LNSCs provides a robust basis for applying modern techniques in future research to identify MHC class loaded peptides on LNSCs by mass spectrometry [57] or reverse immunology using bioinformatics [58].

Next to possible antigen presentation by LNSCs, we demonstrated in this study that human LNSCs possess an arsenal of immunomodulatory molecules both in vitro on cultured human LNSCs and ex vivo on freshly isolated human LNSCs. As previously reported, PD-L1 expression is mostly restricted to endothelial cells (LECs and BECs) on freshly isolated human LN [59]. Similarly, our data also showed that PD-L1 protein expression was higher on endothelial cells in comparison to FRCs and DNs. Interestingly, at mRNA level we could detect low level of PD-L1 and HLA-DR in cultured stromal cells and the expression was lower in RA-risk individuals and RA patients compared with healthy individuals, but future studies are needed to confirm this. Moreover, our data reveal very low levels of co-stimulatory molecules CD80, CD86, and CD40 expression on freshly isolated human LNSCs. This is interesting since tolerogenic DCs possess a low level of costimulatory molecules and therefore can control autoreactive T cells. From these observations we can conclude that human LNSCs have the machinery to interact and influence lymphocytes and therefore to potentially regulate tolerance and adaptive immunity.

Overall, our explorative study shows for the first time citrullination in human LNSCs targeted by autoantibodies isolated from RA patients. Further challenging mechanistic studies are required to investigate whether LNSCs from RA (-risk) patients have an altered tolerogenic effect on autoreactive T cells. To study LNSCs immunoregulatory function, in vitro expansion of LNSCs is required, which is a limitation of this study. The difficulty of obtaining LN biopsies from a large number of individuals and the slow growth rate of human LNSCs limits the number of donors analyzed in this study. In addition, the variation between donors is high. However, by revealing that human LNSCs exhibit the tools to induce tolerance as observed in mice, they become an attractive new therapeutic target to exploit in tolerance maintenance and induction.

## 4. Material and Methods

### 4.1. Study Individuals and Lymph Node Needle Biopsy Sampling

Individuals with arthralgia and elevated IgM-RF and/or ACPA levels, but without any evidence of arthritis upon examination were included (RA-risk individuals, phase c/d upon examination were included (RA-risk individuals, phase c/d; *n*= 23 [8]). Median follow up time of RA-risk individuals was 20.3 months (12.9–33.2 (IQR)) and none of the RA-risk individuals developed arthritis during this period. RA-risk individuals were not allowed to have systemic or intra-articular corticosteroid injection less than 28 days before enrolment. In addition, RA patients with established disease based on fulfillment of the American College of Rheumatology and European League Against Rheumatism (ACR/EULAR 2010 [60] criteria and as assessed by the rheumatologist were included (*n* = 24). Healthy individuals without any joint complaints and without elevated IgM-RF and/or ACPA level and without active viral infection or any history of autoimmunity or malignancy and no present or previous use of disease-modifying antirheumatic drugs (DMARDs), biologicals, or other experimental drugs served as the (voluntary) control group (*n* = 14). IgM-RF was measured using IgM-RF ELISA (Hycor Biomedical, Indianapolis, IN, USA) (ULN (upper limit of normal) 49 kU/mL)). ACPA was measured using anti-CCP2 ELISA CCPlus (Eurodiagnostica, Nijmegen, The Netherlands (ULN 25 kAU/L)). The study was performed according to the principles of the Declaration of Helsinki, approved by the institutional medical ethical review board of the Academic Medical Center (Ethical permission: NL20951.018.07, date: 25 February 2008 and NL52469.018.15, date: 17 July 2015), and all study individuals gave written informed consent. All study individuals underwent an ultrasound-guided inguinal LN needle core biopsy as previously described [61]. At the day of LN sampling none of the donors showed signs of an infection. Table 1 shows the demographics of the included individuals.

### 4.2. Lymph Node Collection and Processing from Kidney Transplantation Recipients

LNs were collected from surgical residual material of kidney transplantation recipients (*n* = 10) during implantation of the kidney. Before anastomosing the arteria and vena renalis, the iliac artery and vein were dissected free. The resulting residual tissue that was removed in this procedure often contains LNs. Though all patients were treated with a quadruple immunosuppressive therapy, only the first dose of CD25mAb was administered before the procedure and we have demonstrated that CD25mAb (basiliximab; Novartis Pharma, Amsterdam, The Netherlands) was not detectable in LN cells and that ex vivo CD25 expression on cells could be blocked with CD25mAb [62]. LNs were carefully cleaned of fat and connective tissue, then cut into small pieces (< 0.5 cm) after which a cell suspension was obtained by grinding the material through a flow-through chamber. The remaining tissue stroma was used in this study and digested as described before [45] to isolate and analyze LNSCs directly ex vivo. In summary, LN stroma was digested using the enzymatic mixture of 0.2 mg/mL collagenase *p* (Roche, Woerden, The Netherlands), 0.8 mg/mL Dispase II (Roche, Woerden, The Netherlands), and 0.1 mg/mL DNAse I (Roche, Woerden, The Netherlands) in RPMI medium (Invitrogen, Landsmeer, The Netherlands) without serum. Cell suspensions were filtered through a 70-μm nylon cell strainer and directly stained for flow cytometry.

### 4.3. Lymph Node Stromal Cell Culture and Stimulation

LNSC culture was performed as previously described [63,64]. In short, after depletion of lymphocytes through a 70 μm cell strainer (BD Falcon, San Jose, CA, USA) the remaining stromal tissue of a freshly collected LN needle core biopsy was plated on a 6-well culture dish (Greiner CELLSTAR^®^, Sigma Aldrich, Zwijndrecht, The Netherlands) (passage 0; P0). Complete cell culture medium was added which consists of Dulbecco’s Modified Eagle Medium (DMEM) low glucose (Gibco, Bleiswijk, The Netherlands) supplemented with 0.1% penicillin (Astellas Pharma Inc, Leiden, The Netherlands), 0.1% streptomycin, 0.05 mg/mL gentamicin, 10 mM HEPES buffer, 2 mM L-glutamine (all Gibco, Bleiswijk, The Netherlands), and 10% fetal calf serum (FCS) (GE Healthcare, Zeist, The Netherlands). To expand cell numbers and for passaging, cultured monolayers of human LNSCs were treated with trypsin (0.05% trypsin/5 mM ethylenediaminetetraacetic acid (Thermo Fisher Scientific, Landsmeer, The Netherlands)) in phosphate buffer saline (PBS, Fresenius Kabi Nederland BV, Zeist, The Netherlands) for 7 min at 37 °C. For harvesting, cells were washed with sterile PBS, trypsinized and the cell suspension was collected and centrifuged for 10 min, 1000 rpm (212 g) at 4 °C. Cells were resuspended in cold complete medium and counted using trypan blue (Sigma Aldrich) in a Bürker-Türk chamber (LO Labor Optik, Lancing, UK). Subsequently, human LNSCs were seeded for different experiments. For flow cytometry LNSCs were plated in a 6-well plate (100,000–200,000/well) and stimulated with 50 ng/mL IFNγ (Affymetrix eBioscience, Landsmeer, The Netherlands). For real-time PCR analysis LNSCs were seeded in a 24-well plate (30,000/well) and stimulated with 10 ng/mL IFNγ. For chamber slides (Thermo Fisher Scientific, Landsmeer, The Netherlands) 5000 LNSCs/well were used. As described previously, this ex vivo LNSC culture model contains a mixture of FRCs and DNs [64].

### 4.4. Immunohistochemical Analysis and (Confocal) Microscopy

LN needle biopsies were snap frozen in Tissue-Tek OCT (Thermo Fisher Scientific, Landsmeer, The Netherlands), cryostat sectioned (7 μm), and stored at −80 °C till further use. Before staining, sections were fixed for 20 min using 2% formaldehyde (Sigma-Aldrich, Zwijndrecht, The Netherlands). Cultured LNSCs were plated out (5000/well) in chamber slides (Thermo Fisher Scientific, Landsmeer, The Netherlands), rested for 24 h in complete medium, washed, fixed with cold methanol (Sigma Aldrich, Zwijndrecht, The Netherlands) for 10 min, and stored at −80 °C till further use. LNSCs on chamber slides were blocked with 1% H_2_O_2_ (Sigma Aldrich, Zwijndrecht, The Netherlands) and 20% human serum (Akademiska pharmacy, Stockholm, Sweden). The following antibodies were generated at the Karolinska Institutet as previously described [35]: human biotinylated monoclonal ACPAs (1325:04C03 = C03, 1325:01B09 = B09) are second generation validated ACPAs as described in Steen et al. [35]. A human biotinylated concentration-matched antibody (E02, reactive against tetanus) was used as a non-ACPA control antibody. Furthermore, in this study rabbit polyclonal anti-PADI2 (Cosmo Bio, Tokyo, Japan), mouse monoclonal anti-PADI4 (Abcam, Cambridge, UK) and corresponding isotype controls were used. Slides were incubated overnight in a moist chamber at 4 °C with the primary/detection antibodies. The next day, slides were first blocked with 1% normal goat serum (Dako, Stockholm, Sweden) and then incubated for 30 min with either biotin-conjugated goat anti-mouse secondary antibody (Invitrogen, Stockholm, Sweden) or a biotin anti-rabbit IgG (H + L) (Vector Laboratories, Stockholm, Sweden). Staining was performed using the VECTASTAIN Elite ABC kit (Vector Laboratories, Stockholm, Sweden) and visualized with 3,3-diaminobenzidine (DAB, Vector Laboratories, Stockholm, Sweden). Slides were counterstained with Mayer’s hematoxylin (Sigma Aldrich Zwijndrecht, The Netherlands), permanently mounted and viewed by a light microscope (Reichert Polyvar 2 type 302001, Leica Microsystems, Wetzlar, Germany). Sections of LN tissue were scored by two independent researchers with a scale ranging from 0 to 4 with 0 = no signal and 4 = very strong signal. For AIRE staining, LNSCs on chamber slides were blocked and permeabilized with PBS buffer containing 3% bovine serum albumin (BSA) and 0.3% Triton (both Sigma Aldrich, Zwijndrecht, The Netherlands) for 30 min and then incubated overnight at 4 °C with monoclonal mouse IgG1 anti-human AIRE antibody (Santa Cruz, Huissen, The Netherlands) or control mouse IgG1 antibody (Dako, Amstelveen, The Netherlands). Subsequently, secondary anti-mouse IgG1-AlexaFluor488 (Invitrogen, Landsmeer, The Netherlands) was used for labelling and chamber slides were mounted using Vectashield Hardset (Vector Laboratories, Stockholm, Sweden). After hardening overnight staining was analyzed by confocal microscope (TCS SP8, Leica Microsystems, Wetzlar, Germany).

### 4.5. Flow Cytometry Analysis

Human LNSCs (passages 5–10) were harvested from a 6-well dish using 1 mL TripLE™ Select (Gibco, Bleiswijk, The Netherlands) for 10 min at 37 °C. Subsequently, cells were washed in PBA buffer (PBS containing 0.01% NaN3 and 0.5% BSA (Sigma Aldrich, Zwijndrecht, The Netherlands), and stained for 1 h with rat IgG2a anti-human Podoplanin (clone NZ-1, AngioBio, Huissen, The Netherlands) on ice. Afterwards cells were washed again in PBS buffer, followed by a second incubation for 30 min on ice protected from light using the following directly labelled antibodies: polyclonal goat anti-rat IgG AlexaFluor647 (Invitrogen, Landsmeer, The Netherlands), CD45 FITC (clone HI30, Becton Dickinson (BD) Pharmingen, Vianen, The Netherlands), CD80 PE (clone 2D10.4, eBioscience, Landsmeer, The Netherlands), CD274 (PD-L1) BV421 (clone MIH1, BD Biosciences, Vianen, The Netherlands), HLA-DR PE-Cy7 (clone L243, Sony Biotechnology, Surrey, UK), and CD40 PE-Cy7 (clone 5C3, Sony Biotechnology, Surrey, UK) or with corresponding isotype control antibodies. Staining with HLA-ABC PE-Cy7 (clone G46–2.6, Biolegend, London, UK) served as a positive control and was used to set-up the correct compensation configuration settings. To assess the HLA-DR and co-stimulatory expression on human LNSCs directly ex vivo, freshly isolated cells were stained with eBioscience™ Fixable Viability Dye eFluor™ 780 (Invitrogen, Landsmeer, The Netherlands) for 15 min, followed by 10 min blocking in PBS containing 5% normal human serum and 2% FCS (Biowest) on ice in the dark. The cells were then incubated with CD45 eFlour 450 (MI30, Invitrogen, Landsmeer, The Netherlands), CD11c Alexa 700 (Bu15, Biolegend, London, UK), HLA-DR PE (clone L243, Invitrogen, Landsmeer, The Netherlands), CD80 Pe-Cy7 (clone 2D10, Biolegend, London, UK), CD86 Pe-Cy5 (clone IT2.2, Biolegend, London, UK), CD40 PerCP/Cy5.5 (clone 5C3, Biolegend, London, UK), PD-L1 BV711 (clone 29E.2A3, Biolegend, London, UK) antibodies for 30 min on ice in the dark. For staining of AIRE protein in LNSCs we used the Fixation/Permeabilization Solution Kit (BD Biosciences, Vianen, The Netherlands) to detect intranuclear expression or the Foxp3/Transcription Factor Staining Buffer Set (eBioscience, Landsmeer, The Netherlands) according to the manufacturer’s instructions. Cells were stained with AIRE PE (clone 614530, R&D Systems, Minneapolis, MN, USA) and mouse IgG1 isotype control (eBioscience, Landsmeer, The Netherlands) for 30 min at RT. Cells were measured on a FACS CANTO II (BD, Vianen, The Netherlands) or a BD LSRFortessa™ X-20 (BD Biosciences, Vianen, The Netherlands) and analyzed with FlowJo software (TreeStar Inc., Ashland, OR, USA).

### 4.6. Quantitative Real-Time PCR and Conventional PCR

Total RNA was isolated using the RNeasy Mini kit or RNeasy Micro kit (Qiagen, Venlo, The Netherlands) according to the manufacturer’s instructions, including a DNAse step to remove genomic DNA. Subsequently cDNA was prepared using the RevertAid H Minus First Strand cDNA Synthesis kit (Thermo Fisher Scientific, Landsmeer, The Netherlands). Quantitative PCR was performed using either Taqman^®^ Universal PCR master mix combined with Taqman assays or SYBR^®^ Green PCR master mix (all from Applied Biosystems, Life Technologies, Zwijndrecht, The Netherlands) combined with in house designed primers (Thermo Fisher Scientific, Landsmeer, The Netherlands). Taqman assays and primer sequences are described in Table S1. For detection we used a StepOnePlus™ Real-Time PCR System or the QuantStudio 3 (Applied Biosystems, Life Technologies, Zwijndrecht, The Netherlands). Values for each target gene were normalized by the expression level of 18S RNA. An arbitrary calibrator sample was used to correct for inter-plate differences. For calculating the relative quantity (RQ) the delta-delta Ct method was used for Taqman assays and a standard curve method was applied for SYBR green assays. An arbitrary calibrator sample was used to correct for inter-plate differences. The fold induction was calculated using the following formula: (RQ stimulated / RQ unstimulated).

For conventional PCR we used the GoTaq DNA, GoTaq green reaction buffer and dNTPs (Promega, Leiden, The Netherlands), and the Biometra T-gradient Thermoblock (Analytic Jena, Jena, Germany) with the following program: denaturation step 3 min at 95 °C, then cycle of 40 times of 30 s 95 °C followed by 30 s at 61 °C and elongation for 45 s at 72 °C and finally 2 min at 72 °C. As positive controls we used an in house made arbitrary RNA sample containing a mixture of RNA isolated from all human tissues (kindly provided by Dr. Huitinga from The Netherlands Institute for Neuroscience) as well as RNA from human thymus tissue (kindly provided by the Pathology department of the AMC, Amsterdam, The Netherlands). Samples were loaded on a 1.5% agarose gel (Invitrogen, Landsmeer, The Netherlands) and mRNA expression was visualized using gel imager Gene Flash (Syngene, Amsterdam, the Netherlands).

### 4.7. Western Blot

Approximately 300,000 LNSCs were collected in 50 µL of radioimmunoprecipitation assay (RIPA) buffer (Bioke, Leiden, The Netherlands) supplemented with leupeptin (1 µL/mL, Sigma Aldrich, Zwijndrecht, The Netherlands), pepstatin A (1 µL/mL, Sigma Aldrich, Zwijndrecht, The Netherlands), and PMSF (phenylmethanesulfonyl fluoride, 4 µL/mL, Sigma Aldrich, Zwijndrecht, The Netherlands) and were stored at −80 °C till further use. After thawing, protein concentration was measured using Pierce BCA protein assay kit (Thermo Fisher Scientific, Landsmeer, The Netherlands). Then, 10 µg of each sample, diluted in NuPAGE reducing agent and NuPAGE LDC buffer (Thermo Fisher Scientific, Landsmeer, The Netherlands) was loaded into a NuPAGE 4–12% gradient gel together with a Protein Molecular Weight Marker (Odyssey^®^ One-Color, LI-COR, Bad Homburg, Germany). Gel was run in MES buffer (Thermo Fisher Scientific, Landsmeer, The Netherlands) for 1 h at 200 V. Subsequently, proteins were transferred onto a PVDF membrane (Immobilon-FL, Merck Millipore, Amsterdam, The Netherlands) for 70 min at 30 V. For washing TBST buffer (50 mM Tris, 150 nM NaCl, 0.1% Tween 20) was used and after blocking of membrane with 5% Blotting-Grade Blocker (Bio-Rad, Veenendaal, The Netherlands), the membrane was incubated with monoclonal mouse IgG1 anti human AIRE (1:500) or polyclonal goat IgG anti human Actin (1:2000) (both Santa Cruz, Huissen, The Netherlands) overnight at 4 °C. The next day the membrane was washed and incubated with anti-mouse or anti-rabbit secondary antibodies conjugated with horseradish peroxidase (HRP) (both 1:2000, both Dako, Stockholm, Sweden) and were visualized using the HRP substrate Lumi-Light Plus (Roche, Woerden, The Netherlands). Blot was analyzed using ImageQuant LAS4000 (GE Healthcare, Zeist, The Netherlands).

### 4.8. Statistics

Data are presented as median with interquartile range (IQR) or mean with standard deviation when normally distributed. Differences between the study groups were analyzed using Kruskal–Wallis test followed by a post-hoc Dunn’s test or a two-way ANOVA test followed by Dunnett’s multiple comparison test, where appropriate. GraphPad Prism software (V.7.01, La Jolla, CA, USA) was used for statistical analysis. *p*-values < 0.05 were considered statistically significant.

## Figures and Tables

**Table 1:** Demographic data of study participants.

Variables	Healthy	RA-Risk	RA
Sex (female) (*n*) (%)	9 (64)	20 (87)	17 (70)
Age (years) (median (IQR))	29 (26–37) ^†^	49 (35–57)	56 (44–61)
IgM-RF positive (*n*) (%)	0 (0)	10 (43)	20 (3–107)
IgM-RF level (kU/mL) (median (IQR))	—	20 (3–107)	131 (31–309)
ACPA positive (*n*) (%)	0 (0)	13 (57)	18 (75)
ACPA level (kAU/L) (median (IQR))	—	43 (4–177)	115 (21–924)
IgM-RF and ACPA both positive (*n*) (%)	0 (0)	0 (0)	14 (58)
DAS28 (median (IQR))	—	—	5 (1–10) ^a,b^
ESR (mm/h) (median (IQR))	—	7 (2–10)	11 (5–27) ^c^
CRP (mg/L) (median (IQR))	0.5 (0.3–1.2) ^a^	1.6 (0.9–3.2)	4.6 (1.4–13) ^d^
68TJC (median (IQR))	0 (0)	1.5 (0–4.5)	9 (4–20) ^e^
68SJC (median (IQR))	0 (0)	0 (0)	5 (1–10) ^d^
Treatment (*n*) (%)			9 (39)
Corticoids			6 (26)
NSAID			4 (17) ^f^
DMARD			5 (22)
Failed TNF inhibitor therapy			5 (22)

IgM-RF, IgM rheumatoid factor; ACPA, anti-citrullinated protein antibodies; ESR, erythrocyte sedimentation rate; CRP, C-reactive protein; TJC, tender joint count; NSAID, non-steroidal anti-inflammatory drug; DMARD, disease-modifying antirheumatic drugs. ^a^ levels missing from one individual, ^b^ levels missing from two individuals, ^c^ levels missing from six individuals, ^d^ levels missing from seven individuals, ^e^ levels missing from five individuals, ^f^ treatment unknown for five individuals. ^†^ Healthy individuals are significantly younger than RA-risk individuals and RA patients (*p* < 0.0050, tested by Kruskal–Wallis followed by a post Dunn’s test).

## Supplementary Materials

The following are available online at https://www.mdpi.com/1422-0067/21/16/5713/s1. Figure S1: Intranuclear staining of Aire protein in cultured human LNSCs; Figure S2: Expression of RRAD and GAD1 stratified according to ACPA status; Figure S3: Induction of IFNGR1 after stimulation with IFNγ in human LNSCs; Table S1: Primers used in this study.
