# Supplementary material for: Human Lymph Node Stromal Cells Have the Machinery to Regulate Peripheral Tolerance during Health and Rheumatoid Arthritis
Source: Int J Mol Sci. 2020 Aug 9;21(16):5713. doi: 10.3390/ijms21165713 (PMC7460812; doi:10.3390/ijms21165713)
Supplement: Supplementary file 1 [file ijms-21-05713-s001.pdf]

International Journal of
Molecular Sciences

MDPI

Supplementary Materials:

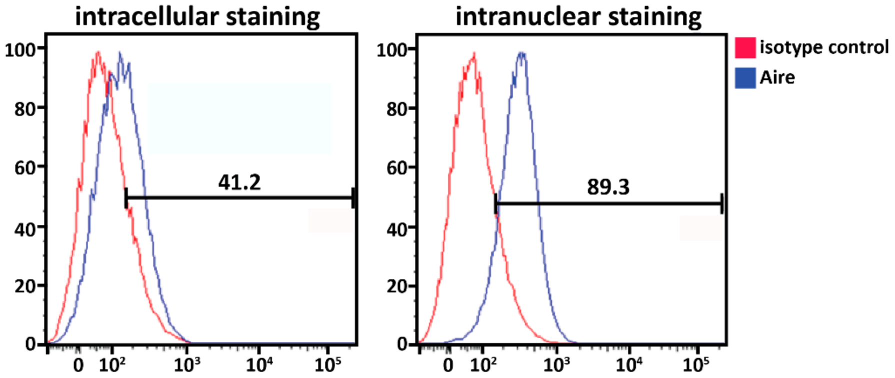

Figure S1. Intranuclear staining of Aire protein in cultured human LNSCs. Aire protein expression was measured by flow cytometry comparing intracellular versus intranuclear staining (tested on 2 healthy donors passage 3). Histogram presents % of positive cells in comparison to isotype staining.

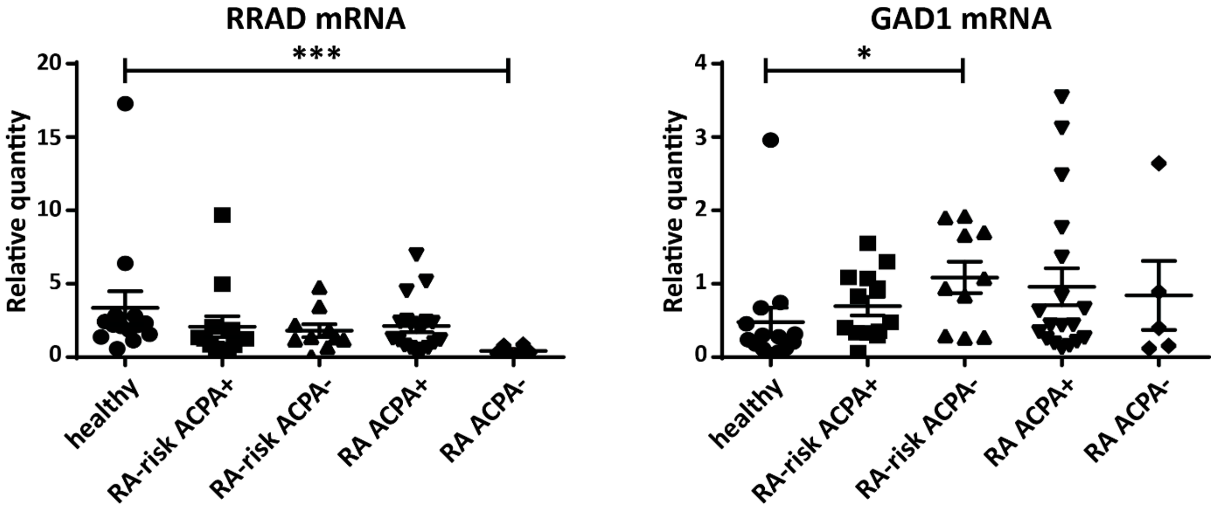

Figure S2. Expression of RRAD and GAD1 stratified according to ACPA status. Expression of RRAD and GAD1 was assessed by qPCR in LNSCs of passage 2 derived from different donor groups (healthy individuals $n = 14$, RA-risk ACPA+ individuals $n = 13$, RA-risk ACPA− individuals $n = 10$, RA ACPA+ patients $n = 18$ and RA ACPA− patients $n = 6$). Relative quantity is displayed as median and interquartile range. Differences between donor groups were assessed by Kruskal-Wallis followed by a post Dunn's test. *$p < 0.050$, **$p < 0.010$, ***$p < 0.001$.

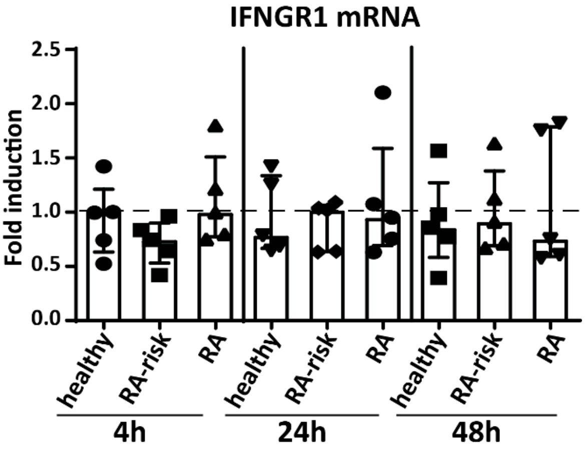

Figure S3. Induction of IFNGR1 after stimulation with IFNγ in human LNSCs. Expression of IFNγR1 was assessed by qPCR in LNSCs after stimulation with IFNγ for 4 h, 24 h or 48 h. Data are represented as fold induction (median with interquartile range) by comparing the protein levels in stimulated cells to corresponding unstimulated cells in 15 donors (*n* = 5 per donor group). Dotted line represents a fold induction value of 1.

Table S1. Primers used in this study.

**Gene symbol**	**Taqman Assay ID**	
DEAF1	Hs00221402_m1	
AIRE	Hs00230829_m1	
HLA-DR	Hs00219575_m1	
CD80	Hs00175478_m1	
CD86	Hs01567026_m1	
IL-10	Hs00961622_m1	
CD274	Hs01125301_m1	
NOS2	Hs01075529_m1	
18S	Hs99999901_s1	
**Gene symbol**	**Forward primer sequence 5′-3′**	**Reverse primer sequence 5′-3′**
RRAD	GCAGCAGGGCACACCTATGA	CCGTCCTGCTCCCAAATGTC
GAD1	CCCACAACGTACGATACCTG	CACAAGGCGACTCTTCTCTT
PLP1	TCAATGTGATCCATGCCTTCCA	GGTGGTCTTGTAGTCGCCAA
AFP	CGAACTTTCCAAGCCATAACTG	CTCCTGGTATCCTTTAGCAACT
IFNGR1	CATCACGTCATACCAGCCATTT	CTGGATTGTCTTCGGTATGCAT
CD40	CACCTCGCTATGGTTCGTCT	GGCACAAAGAACAGCACTGA
TGFB1	TGACCTGGCCACCATTCAT	TCCGTGGAGCTGAAGCAATAG
IDO	TTGCTAAAGGCGCTGTTGGA	TGCCTTTCCAGCCAGACAAAT
18S	CCGAGTAAGTGCGGGTCATAA	CCATCCAATCGGTAGTAGCG
